# The Association of *ERCC1* and *ERCC5* Polymorphisms with Lung Cancer Risk in Han Chinese

**DOI:** 10.7150/jca.59277

**Published:** 2022-01-01

**Authors:** Xueling Lan, Ying Li, Yefeng Wu, Xia Li, Lan Xu

**Affiliations:** 1 Department of Laboratory, Cancer Hospital of China Medical University, Liaoning Cancer Hospital & Institute, Shenyang, Liaoning 110042, China.; 2 Department of Radiation Oncology, Cancer Hospital of China Medical University, Liaoning Cancer Hospital & Institute, and Key Laboratory of Tumor Radiosensitization and Normal Tissue Radioprotection of Liaoning Province, Shenyang, Liaoning 110042, China.; 3 Central Laboratory, Cancer Hospital of China Medical University, Liaoning Cancer Hospital & Institute, Shenyang, Liaoning 110042, China.

**Keywords:** ERCC, polymorphisms, lung cancer, risk

## Abstract

**Background:** Polymorphisms in DNA damage repair genes are important determinants for cancer susceptibility, clinical phenotype diversity, and therapy. However, their relationship with lung cancer remains unclear. This study aimed to investigate the role of DNA damage repair gene polymorphisms in the risk of lung cancer.

**Methods:** The matrix-assisted laser desorption ionization-time of flight (MALDI-TOF) mass spectroscopy-based genotyping system was used to genotype 601 individuals (200 lung cancer patients and 401 age- and sex-matched healthy controls) for polymorphisms in excision repair cross-complementing group 1 (*ERCC1*) and *ERCC5* genes.

**Results:** The *ERCC5* rs4771436 GG genotype, recessive model (GG vs. GT+TT), and the *ERCC5* rs1047768 recessive model (CC vs. CT+TT) were associated with significantly increased risks of lung cancer (*P*=0.029, *P*=0.014, and *P*=0.044, respectively), especially in men and individuals aged 60 years or younger.

**Conclusion:** *ERCC5* rs4771436 and rs1047768 genotypes were associated with an increased risk of lung cancer, suggesting that polymorphisms in DNA repair genes are significantly related to the risk of lung cancer, and play an important role in the occurrence of lung cancer.

## Introduction

Lung cancer is one of the common malignant tumors in the world, and it remains the leading cause of cancer mortality because of its high malignant and metastatic potential 1. Epidemiological studies of migrant populations point to a role for environmental and/or lifestyle factors in cancer etiology 2-6. The occurrence of lung cancer is closely related to smoking, as shown by its observed downward trend in global incidence with the launch of anti-smoking campaigns; however, it still ranks first among all cancer types. In recent years, in addition to environmental factors, genetic factors have become a hot spot in the etiology of lung cancer.

Alterations in the DNA damage repair pathway are hallmarks of cancer 7, and the relationships between such pathways and cancer are varied and complex. DNA repair pathways are essential for preventing DNA damage from causing mutations and cytotoxicity 8, but the incorrect repair of DNA lesions often leads to carcinogenesis and genomic instability 7. An important connection linking the DNA damage repair pathway to cancer development is variations in DNA damage repair genes.

Single nucleotide polymorphisms (SNPs) are the most common type of genetic variation, and participate in carcinogenesis. SNPs in genes encoding proteins involved in DNA damage repair pathways are associated with the risk and prognosis of various cancers, including lung cancer. For example, the X-ray repair cross-complementing protein 1 gene (*XRCC1*) codon 399 Gln allele and *TP53* codon 72 Arg allele appear to have a protective effect against lung adenocarcinoma, especially in individuals older than 50 years of age 9. Moreover, excision repair cross-complementing group 1 gene (*ERCC1*) rs3212986 GG homozygosity and rs11615 T allele were associated with a higher risk of developing non-small cell lung cancer (NSCLC) in the Polish population 10. *ERCC2* rs13181 and *ERCC1* rs3212986 SNPs have an elevated association with lung cancer risk 2, 11, while the O6-methylguanine-DNA methyltransferase gene SNP rs12917 is associated with an increased risk of lung cancer 12. Additionally, the *ERCC2* rs13181 TG genotype and rs1799793 CT genotype significantly increased the risk of cancer death 13. The identification of these SNPs could be a useful low-cost tool for evaluating individual cancer risk, promoting the earlier detection and management of cancer.

A complex DNA repair machinery has evolved to protect genomic integrity in the face of a myriad of DNA damage sources. If DNA repair fails, this damage can lead to carcinogenesis and tumor genomic instability 14. Genetic and epigenetic aberrations in DNA damage repair pathway genes are associated with various pathogeneses 15-22. These changes may be useful biomarkers in a liquid biopsy for the early detection and prevention of lung cancer. Here, we investigated the link between SNPs in DNA damage repair pathway genes and susceptibility to lung cancer by studying three ERCC1 and two ERCC5 SNPs in a Chinese Han population.

## Materials and Methods

### Study design and study population

This study design was approved by the Human Ethics Committee of Liaoning Cancer Hospital (Shenyang, China). Each participant provided their written informed consent during an epidemiological investigation. A total of 200 lung cancer patients were recruited from Liaoning Cancer Hospital who had undergone surgical resection or needle biopsy diagnosis/treatment between 2018 and 2019. A total of 401 age- and sex-matched healthy controls were recruited from a health check program in Liaoning Province between 2018 and 2019. All diagnoses were based on histopathological examinations. Information about smoking status, alcohol consumption, and family history were acquired in a face-to-face questionnaire survey. Fasting venous blood was obtained from participants and stored at -20 °C.

To evaluate the relationship between SNPs and clinicopathological parameters of lung cancer, histology or clinical data were assessed according to World Health Organization criteria, and tumor-node-metastasis (TNM) staging was performed according to the 8^th^ edition of the International Union against Cancer/American Joint Committee on Cancer 2017 criteria 23.

### SNP selection

A compilation of the genes involved in the DNA damage repair pathway was conducted on the basis of a published panel of DNA damage repair genes 24-27 and NCBI-Gene website analysis (https://www.ncbi.nlm.nih.gov/gene/). We selected the following five SNPs for analysis in this study: *ERCC1* rs735482, rs11615, and rs3212986 and *ERCC5* rs4771436 and rs1047768.

### SNP genotyping

Genomic DNA was extracted from peripheral blood samples obtained from study participants using the phenol/chloroform method according to our standard procedure 28. Matrix-assisted laser desorption ionization-time of fight (MALDI-TOF) mass spectroscopy-based genotyping was used to genotype all 601 individuals for SNPs in the five DNA damage repair genes.

### Statistical analysis

Statistical analysis was performed using SPSS statistical software (version 22.0). Adjusted odds ratios and 95% confidence intervals (CIs) for the relationship between SNPs and lung cancer risk were calculated by multivariable logistic regression, with adjustment for sex and age. In the analysis stratified by sex, the age was adjusted and vice versa. The χ^2^ test was used to evaluate the relationship between polymorphism genotypes and the clinicopathological parameters of lung cancer patients. Logistic regression was used for the interaction and epistatic effect analysis of ERCC1 and ERCC5 polymorphisms in the risk of lung cancer. Haplotype-base risk prediction of SNPs in ERCC1 and ERCC5 genes for lung cancer was performed using the HaploView (https://www.broadinstitute.org/scientific-community/science/programs/medical-and-population-genetics/haploview/haploview).

## Results

### Baseline patient characteristics

A comparison of baseline characteristics is shown in Table 1. There was a significant difference in age distribution between lung cancer patients and controls, but not with respect to sex. The mean age and mean age of menarche also differed significantly between patients and controls (both *P* < 0.001). The mean menopausal age in patients was 60.50 years and only a small proportion had a family history of cancer (14.1%). Regarding tumor invasion depth, 45.8% and 54.2% of patients were in T1-2 and T3-4, respectively. Tumor stages I-II (10.1%) and III-IV (89.9%) accounted for most lung cancer cases, and 80.5% of patients had positive lymph nodes while 63.6% had metastasis.

### Association of *ERCC1* and *ERCC5* SNPs with lung cancer risk

Multivariable logistic regression was used to investigate the association of *ERCC1 a*nd *ERCC5* SNPs with lung cancer risk. *ERCC5* rs4771436 and rs1047768 had a significant association with lung cancer risk progression (Table 2). Specifically, we found that carriers of the *ERCC5* rs4771436 GG genotype, the recessive model (GG vs. GT+TT) and the ERCC5 rs1047768 CC genotype, the recessive model (CC vs. CT+TT) showed a significantly increased risk of lung cancer (*P*<0.05). However, there was no significant association between *ERCC1* SNPs and lung cancer risk progression.

### Stratified analysis of *ERCC1* and *ERCC5* SNPs with lung cancer risk

Using stratified analysis, we showed that the *ERCC5* rs4771436 GG genotype, the recessive model (GG vs. GT+TT) and *ERCC5* rs1047768 CC genotype, the recessive model (CC vs. CT+TT) conferred 5.01-fold, 5.39-fold, 3.06-fold, and 3.25-fold increases in lung cancer progression, respectively, in patients aged ≤60 years. In older individuals (aged >60 years), no genotype was significantly correlated with the risk of lung cancer. In men, the *ERCC5* rs1047768 the recessive model (CC vs. CT+TT) conferred a 3.00-fold increase in lung cancer progression. However, no SNPs were significantly associated with the risk of lung cancer in women. These results are shown in Table 3.

### Association of *ERCC1* and *ERCC5* SNPs with clinicopathological parameters of lung cancer patients

Among the SNPs associated with an increased risk of lung cancer, *ERCC1* rs735482 in the recessive model was significantly related to pathological type. Moreover, the heterozygous genotype of *ERCC1* rs11615 and *ERCC5* rs1047768 in the recessive model were significantly related to sex, while the heterozygous genotype and *ERCC5* rs4771436 in the dominant model and *ERCC5* rs1047768 in the recessive model were significantly related to smoking. Other SNPs had no significant correlation with clinicopathological parameters. All results are shown in Table 4.

### The interaction and epistatic effect analysis and HaploView in the risk of lung cancer

In the logistic regression analysis, the interaction and epistatic effects were not found, and all results are shown in Table 5 and Table 6. Haplotype-base risk prediction of SNPs in ERCC1 and ERCC5 genes for lung cancer was performed using the HaploView. ERCC1 rs4771436 and rs1047768 were highly linked, and ERCC5 rs735482 and rs11615, rs3212986 and rs11615 were also highly linked, forming haplotype blocks (D' >0.95). Haplotype block were T-C, G-T, T-T, C-A, A-A, C-G, A-G, C-A, A-G, C-G, respectively. There were no significant statistical differences in this analysis. All results were presented in Table 7.

## Discussion

DNA damage repair pathways play an important role in the occurrence and development of cancer, especially in lung cancer which has high morbidity and mortality. Cancer cells carry various types of mutations and show the aberrant expression of genes involved in DNA repair responses, leading to genome instability, the promotion of carcinogenesis, and cancer progression. Defects in DNA repair responses have been considered suitable biomarkers for cancer risk screening 29. The association of *ERCC* genetic variation with lung cancer has been widely evaluated worldwide 17, 30, but has been rarely reported in the Han Chinese population, especially in Liaoning Province.

*ERCC* polymorphisms are also known to be closely related to the occurrence and development of other cancers. For instance, *ERCC3* rs4150434 and *ERCC5* rs4771436 and rs2094258 SNPs were previously associated with genetic susceptibility to lung cancer 31, *ERCC5* rs2296147 was associated with a reduced risk of esophageal cancer 32, and *ERCC2* rs1799793 was positively associated with prostate cancer risk in an Asian population 16. Moreover, five SNPs (rs1047768, rs2227869, rs1047768, rs17655, and rs2227869) of *ERCC5*, a gene involved in nucleotide excision repair, were associated with a reduced stomach cancer risk 33.

Of course, there are also genetic polymorphisms that affect the risk of lung cancer by affecting *ERCC* mutations, such as rs229614 and rs17655, which may be one of the molecular mechanisms of lung cancer 30. Other polymorphisms are also significantly associated with the risk of lung cancer; for example those in* XRCC1* and *TP53*, especially in individuals aged over 50 years, whose detection allows the earlier diagnosis of disease 9. *ERCC1* and *XRCC1* polymorphisms have also been significantly associated with the risk of lung cancer, especially in non-smokers 2-5. Additionally, Chaszczewska et al. reported that a nuclear factor kappa B subunit 2 polymorphism may be associated with NSCLC risk in the Polish population, and is a potential marker for NSCLC in men 10. Moreover, a *XRCC1* polymorphism was closely related to the incidence of NSCLC, especially in women 3. In the high incidence region of Hebei Province, the C/C genotype of *XPC* exon 15 appears to increase the risk of developing esophageal squamous cell carcinoma in the non-smoking population 6. Polymorphisms in DNA repair genes may be related to an increased risk of malignant transformation in lung cancer, especially among smokers and residents of coal mining areas 34.

Our findings suggest that *ERCC5* might be a candidate gene for lung cancer susceptibility in the Han Chinese population. We report for the first time a significant association between *ERCC5* SNPs rs4771436 and rs1047768 with lung cancer risk progression in Liaoning Province. We found that carriers of the *ERCC5* rs4771436 GG genotype, the recessive model (GG vs. GT+TT) and the *ERCC5* rs1047768 CC genotype, the recessive model (CC vs. CT+TT) had increased risks of lung cancer. Our findings provide experimental evidence to support the use of *ERCC1* and *ERCC5* SNPs as potential biomarkers of specific types of lung cancer.

We conducted stratified analyses in our study to examine how age and sex affected the correlation between SNPs and the risk of lung cancer. We found that the *ERCC5* rs4771436 GG genotype, the recessive model (GG vs. GT+TT) and the *ERCC5* rs1047768 CC genotype, the recessive model (CC vs. CT+TT) conferred increases in lung cancer progression in individuals aged ≤60 years. Additionally, the *ERCC5* rs1047768 the recessive model (CC vs. CT+TT) conferred an increase in lung cancer progression in men. These results are consistent with reported findings, although potential underlying mechanisms require further investigation.

Liu et al. previously detected a correlation between the tumor stage of lung cancer patients and *ERCC1* SNP rs3212986 5. Furthermore, the tumor necrosis factor receptor superfamily, member 19 gene plays an inhibitory role in lung cancer, and its differential expression is significantly related to tumor TNM staging 35. Clinicopathological parameters such as age, sex, smoking status, and tumor stage are associated with the distribution of genetic polymorphisms and the risk of tumor incidence. In the present study, we compared the genotype distribution of the five SNPs in lung cancer patients with different clinicopathological parameters. We found that *ERCC1* rs735482 in the recessive model was significantly related to pathological type, being least common among patients with squamous cell carcinoma. Moreover, the heterozygous genotype of *ERCC1* rs11615 and *ERCC5* rs1047768 in the recessive model were significantly related to sex, with the heterozygous *ERCC1* rs11615 genotype being most widely distributed among men and the mutation genotype of *ERCC5* rs1047768 least common among women. Finally, the heterozygous genotype of *ERCC5* rs4771436 and this SNP in the dominant model together with *ERCC5* rs1047768 in the recessive model were significantly related to smoking. Other SNPs had no significant correlation with clinicopathological parameters. Because these results derived from a correlation study, they should be confirmed by conducting basic experiments.

In addition, we have further done SNPs-SNPs interaction, epistatis effect and haplotype analysis. ERCC1 and ERCC5 are located on chromosome 13 and chromosome 19, respectively. ERCC1 rs4771436 and rs1047768 were highly linked, and ERCC5 rs735482 and rs11615, rs3212986 and rs11615 were also highly linked, forming haplotype blocks (D' >0.95). Haplotype block were T-C, G-T, T-T, C-A, A-A, C-G, A-G, C-A, A-G, C-G, respectively. However, there were no significant statistical differences.

Some limitations should be considered in our study. First, the sample size was relatively small, especially of lung cancer patients, so our findings need further confirmation in larger populations. Second, we only analyzed the risk of lung cancer, yet prognostic parameters such as overall survival and progression-free survival also warrant additional study. Finally, functional experiments are required to elucidate the underlying disease mechanisms.

Taken together, our results indicate that *ERCC5* SNPs have a significant association with lung cancer risk progression. *ERCC5* rs4771436 and rs1047768 were found to increase lung cancer risk, especially in men or those aged ≤60 years. These correlations appear to be explained by the distribution of individual SNPs in patients with different clinicopathological parameters. It is to be expected that data from a larger population sample will support these findings, which could then be used to guide the clinical treatment of lung cancer.

## Figures and Tables

**Table 1:** The baseline characteristics of the objects

Characteristics	Cases	Controls	*P* value
Sample size	200	401	
**Age**			<0.001
Mean±SD	58.76±9.60	36.25±12.63	
Mmenarche	60.5	32	
Range	27-80	17-73	
**Gender**			
Female	75 (37.5%)	175 (43.6%)	0.150
Male	125 (62.5%)	226 (56.4%)	
**T stage**			
1-2	60 (45.8%)		
3-4	71 (54.2%)		
**N stage**			
Negative	29 (19.5%)		
Positive	120 (80.5%)		
**M stage**			
Negative	71 (36.4%)		
Positive	124 (63.6%)		
**Clinical stage**			
I-II	20 (10.1%)		
III-IV	178 (89.9%)		
**Smoking**			
No	117 (58.5%)		
Yes	83 (41.5%)		
**Drinking**			
No	42 (21.0%)		
Yes	158 (79.0%)		
**Family history of cancer**			
No	170 (85.9%)		
Yes	28 (14.1%)		
**Pathological type**			
Small cell cancer	57 (30.0%)		
Squamous carcinoma	37 (19.5%)		
Adenocarcinoma	96 (50.5%)		
**Ki67**			
≤50	5 (26.3%)		
>50	14 (73.7%)		
**EGFR**			
Wild type	19 (35.8%)		
Mutation type	34 (64.2%)		
**SCC**			
Normal	57 (78.1%)		
Increased	16 (21.9%)		
**CEA**			
Normal	95 (50.3%)		
Increased	94 (49.7%)		
**CYFRA**			
Normal	68 (43.6%)		
Increased	88 (56.4%)		
**NSE**			
Normal	35 (23.2%)		
Increased	116 (76.8%)		
**PRO**			
Normal	22 (45.8%)		
Increased	26 (54.2%)		
**TAP**			
Normal	4 (8.7%)		
Increased	42 (91.3%)		
**TK1**			
Normal	5 (71.4%)		
Increased	2 (28.6%)		

**Table 2:** The association of ERCC1 and ERCC5 polymorphisms with lung cancer risk

Genetype	SNP	Cases	Controls	*P* value	*P* value	OR (95%CI)
ERCC1	rs735482	N=199	N=400	0.367		
	AA	61(30.5%)	124(31.0%)		/	1(Ref)
	CA	107(53.5%)	196(49.0%)		0.161	1.49(0.85,2.58)
	CC	31(15.5%)	80(20.0%)		0.537	0.79(0.38,1.66)
	CA+CC vs. AA	/	/		0.375	1.27(0.75,2.14)
	CC vs. CA+AA	/	/		0.153	0.62(0.32,1.19)
ERCC1	rs11615	N=200	N=400	0.299		
	AA	18(9.0%)	24(6.0%)		/	1(Ref)
	GA	67(33.5%)	151(37.8%)		0.620	0.77(0.28,2.16)
	GG	115(57.5%)	225(56.3%)		0.946	0.97(0.36,2.60)
	GA+GG vs. AA	/	/		0.799	0.88(0.34,2.32)
	GG vs. GA+AA	/	/		0.507	1.18(0.72,1.92)
ERCC1	rs3212986	N=199	N=396	0.809		
	CC	95(47.7%)	187(47.2%)		/	1(Ref)
	CA	83(41.7%)	173(43.7%)		0.993	1.00(0.60,1.66)
	AA	21(10.6%)	36(9.1%)		0.812	1.11(0.48,2.55)
	CA+AA vs. CC	/	/		0.942	1.02(0.63,1.65)
	AA vs. CA+CC	/	/		0.799	1.11(0.51,2.45)
ERCC5	rs4771436	N=198	N=396	0.616		
	TT	104(52.5%)	207(52.3%)		/	1(Ref)
	GT	78(39.4%)	165(41.7%)		0.498	0.84(0.50,1.40)
	GG	16(8.1%)	24(6.1%)		**0.029**	2.89(1.11,7.53)
	GT+GG vs. TT	/	/		0.951	1.02(0.63,1.64)
	GG vs. GT+TT	/	/		**0.014**	3.25(1.26,8.36)
ERCC5	rs1047768	N=200	N=396	0.391		
	TT	105(52.5%)	197(49.7%)		/	
	CT	72(36.0%)	163(41.2%)		0.181	0.70(0.41,1.18)
	CC	23(11.5%)	36(9.1%)		0.105	2.09(0.86,5.08)
	CT+CC vs. TT	/	/		0.550	0.86(0.53,1.40)
	CC vs. CT+TT	/	/		**0.044**	2.40(1.02,5.61)

**Table 3:** Stratified analysis of the association of ERCC1 and ERCC5 polymorphisms with lung cancer risk

Genetype	SNP	Cases	Controls	*P* value	*P* value	OR (95%CI)
**Age >60**						
ERCC1	rs735482	N=100	N=17	0.499		
	AA	30(25.6%)	6(5.1%)		/	1(Ref)
	CA	50(42.7%)	6(5.1%)		0.348	1.82(0.52,6.39)
	CC	20(17.1%)	5(4.3%)		0.809	0.85(0.22,3.22)
	CA+CC vs. AA	/	/		0.558	1.39(0.46,4.20)
	CC vs. CA+AA	/	/		0.378	0.59(0.18,1.90)
ERCC1	rs11615	N=100	N=17	0.360		
	AA	11(9.4%)	1(0.9%)		/	1(Ref)
	GA	37(31.6%)	4(3.4%)		0.808	1.36(0.11,16.18)
	GG	52(44.4%)	12(10.3%)		0.424	0.41(0.05,3.62)
	GA+GG vs. AA	/	/		0.610	0.57(0.07,4.88)
	GG vs. GA+AA	/	/		0.139	0.43(0.14,1.32)
ERCC1	rs3212986	N=100	N=17	0.821		
	CC	55(47.0%)	8(6.8%)		/	1(Ref)
	CA	36(30.8%)	7(6.0%)		0.538	0.70(0.23,2.16)
	AA	9(7.7%)	2(1.7%)		0.553	0.59(0.10,3.37)
	CA+AA vs. CC	/	/		0.481	0.69(0.24,1.95)
	AA vs. CA+CC	/	/		0.676	0.70(0.13,3.71)
ERCC5	rs4771436	N=98	N=17	0.388		
	TT	51(44.3%)	9(7.8%)		/	1(Ref)
	GT	43(37.4%)	6(5.2%)		0.503	1.49(0.46,4.82)
	GG	4(3.5%)	2(1.7%)		0.263	0.32(0.05,2.34)
	GT+GG vs. TT	/	/		0.820	1.13(0.39,3.30)
	GG vs. GT+TT	0.1	/		0.189	0.29(0.04,1.86)
ERCC5	rs1047768	N=100	N=17	0.798		
	TT	46(39.3%)	9(7.7%)		/	1(Ref)
	CT	44(37.6%)	6(5.1%)		0.540	1.42(0.46,4.41)
	CC	10(8.5%)	2(1.7%)		0.960	1.05(0.18,5.93)
	CT+CC vs. TT	/	/		0.642	1.28(0.45,3.63)
	CC vs. CT+TT	/	/		0.746	0.76(0.14,4.11)
**Age ≤60**						
ERCC1	rs735482	N=99	N=383	0.126		
	AA	31(6.4%)	118(24.5%)		/	1(Ref)
	CA	57(11.8%)	190(39.4%)		0.279	1.40(0.76,2.60)
	CC	11(2.3%)	75(15.6%)		0.763	0.87(0.35,2.16)
	CA+CC vs. AA	/	/		0.450	1.26(0.69,2.28)
	CC vs. CA+AA	/	/		0.296	0.66(0.30,1.44)
ERCC1	rs11615	N=100	N=383	0.301		
	AA	7(1.4%)	23(4.8%)		/	1(Ref)
	GA	30(6.2%)	147(30.4%)		0.540	0.70(0.23,2.16)
	GG	63(13.0%)	213(44.1%)		0.665	1.31(0.39,4.45)
	GA+GG vs. AA	/	/		0.977	0.98(0.32,3.04)
	GG vs. GA+AA	/	/		0.112	1.58(0.90,2.77)
ERCC1	rs3212986	N=99	N=379	0.397		
	CC	40(8.4%)	179(37.4%)		/	1(Ref)
	CA	47(9.8%)	166(34.7%)		0.764	1.09(0.62,1.93)
	AA	12(2.5%)	34(7.1%)		0.574	1.31(0.52,3.30)
	CA+AA vs. CC	/	/		0.683	1.12(0.65,1.94)
	AA vs. CA+CC	/	/		0.648	1.23(0.51,2.96)
ERCC5	rs4771436	N=100	N=379	0.073		
	TT	53(11.1%)	198(41.3%)		/	1(Ref)
	GT	35(7.3%)	159(33.2%)		0.247	0.71(0.39,1.27)
	GG	12(2.5%)	22(4.6%)		**0.002**	5.01(1.77,14.20)
	GT+GG vs. TT	/	/		0.925	0.97(0.57,1.67)
	GG vs. GT+TT	/	/		**0.001**	5.39(1.99,14.62)
ERCC5	rs1047768	N=100	N=379	**0.042**		
	TT	59(12.3%)	188(39.2%)		/	1(Ref)
	CT	28(5.8%)	157(32.8%)		0.073	0.57(0.31,1.05)
	CC	13(2.7%)	34(7.1%)		**0.034**	3.06(1.09,8.63)
	CT+CC vs. TT	/	/		0.383	0.78(0.46,1.35)
	CC vs. CT+TT	/	/		**0.012**	3.25(1.29,8.19)
**Male**						
ERCC1	rs735482	N=125	N=225	0.104		
	AA	39(11.1%)	76(21.7%)		/	1(Ref)
	CA	67(19.1%)	97(27.7%)		0.264	1.53(0.73,3.22)
	CC	19(5.4%)	52(14.9%)		0.393	0.66(0.25,1.71)
	CA+CC vs. AA	/	/		0.590	1.21(0.61,2.40)
	CC vs. CA+AA	/	/		0.131	0.52(0.22,1.22)
ERCC1	rs11615	N=125	N=225	0.405		
	AA	14(4.0%)	16(4.6%)		/	1(Ref)
	GA	40(11.4%)	79(22.6%)		0.500	0.65(0.19,2.27)
	GG	71(20.3%)	130(37.1%)		0.798	0.86(0.27,2.77)
	GA+GG vs. AA	/	/		0.665	0.78(0.24,2.46)
	GG vs. GA+AA	/	/		0.547	1.22(0.64,2.35)
ERCC1	rs3212986	N=125	N=222	0.981		
	CC	59(17.0%)	105(30.0%)		/	1(Ref)
	CA	56(16.1%)	98(28.2%)		0.965	1.02(0.52,1.98)
	AA	10(2.9%)	19(5.5%)		0.780	1.19(0.35,3.98)
	CA+AA vs. CC	/	/		0.906	1.04(0.55,1.99)
	AA vs. CA+CC	/	/		0.778	1.19(0.36,3.88)
ERCC5	rs4771436	N=124	N=222	0.077		
	TT	75(21.7%)	113(32.7%)		/	1(Ref)
	GT	38(11.0%)	95(27.5%)		0.070	0.51(0.25,1.06)
	GG	11(3.2%)	14(4.0%)		0.186	2.39(0.66,8.73)
	GT+GG vs. TT	/	/		0.234	0.67(0.35,1.29)
	GG vs. GT+TT	/	/		0.063	3.52(0.94,13.22)
ERCC5	rs1047768	N=125	N=223	0.420		
	TT	63(18.1%)	112(32.2%)		/	1(Ref)
	CT	43(12.4%)	87(25.0%)		0.359	0.71(0.34,1.48)
	CC	19(5.5%)	24(6.9%)		0.095	2.54(0.85,7.59)
	CT+CC vs. TT	/	/		0.905	0.96(0.50,1.84)
	CC vs. CT+TT	/	/		**0.042**	3.00(1.04,8.68)
**Female**						
ERCC1	rs735482	N=74	N=175	0.924		
	AA	22(8.8%)	48(19.3%)		/	1(Ref)
	CA	40(16.1%)	99(39.8%)		0.410	1.42(0.62,3.23)
	CC	12(4.8%)	28(11.2%)		0.938	1.05(0.32,3.46)
	CA+CC vs. AA	/	/		0.460	1.36(0.60,3.06)
	CC vs. CA+AA	/	/		0.665	0.80(0.30,2.18)
ERCC1	rs11615	N=75	N=175	0.743		
	AA	4(1.6%)	8(3.2%)		/	1(Ref)
	GA	27(10.8%)	72(28.8%)		0.897	1.13(0.17,7.56)
	GG	44(17.6%)	95(38.0%)		0.798	1.28(0.19,8.44)
	GA+GG vs. AA	/	/		0.838	1.21(0.19,7.55)
	GG vs. GA+AA	/	/		0.748	1.13(0.54,2.34)
ERCC1	rs3212986	N=74	N=174	0.412		
	CC	36(14.5%)	82(33.1%)		/	1(Ref)
	CA	27(10.9%)	75(30.2%)		0.976	0.99(0.45,2.16)
	AA	11(4.4%)	17(6.9%)		0.963	1.03(0.32,3.30)
	CA+AA vs. CC	/	/		0.986	0.99(0.48,2.05)
	AA vs. CA+CC	/	/		0.935	1.05(0.36,3.06)
ERCC5	rs4771436	N=74	N=174	0.099		
	TT	29(11.7%)	94(37.9%)		/	1(Ref)
	GT	40(16.1%)	70(28.2%)		0.323	1.47(0.69,3.14)
	GG	5(2.0%)	10(4.0%)		0.073	3.65(0.89,14.99)
	GT+GG vs. TT	/	/		0.164	1.67(0.81,3.43)
	GG vs. GT+TT	/	/		0.108	3.00(0.78,11.46)
ERCC5	rs1047768	N=75	N=173	0.597		
	TT	42(16.9%)	85(34.3%)		/	1(Ref)
	CT	29(11.7%)	76(30.6%)		0.347	0.70(0.33,1.48)
	CC	4(1.6%)	12(4.8%)		0.654	1.43(0.30,6.77)
	CT+CC vs. TT	/	/		0.447	0.76(0.37,1.55)
	CC vs. CT+TT	/	/		0.561	1.56(0.35,6.89)

**Table 4:** The association of ERCC1 and ERCC5 polymorphisms with clinicopathological parameters of lung cancer patients

	ERCC1 rs735482							ERCC1 rs11615							ERCC1 rs3212986							ERCC5 rs4771436							ERCC5 rs1047768						
Characteristics	Wild	Heterozygous	*P* value	Mutation	*P* value	*P*_dominance_	*P*_recessive_	Wild	Heterozygous	*P* value	Mutation	*P* value	*P*_dominance_	*P*_recessive_	Wild	Heterozygous	*P* value	Mutation	*P* value	*P*_dominance_	*P*_recessive_	Wild	Heterozygous	*P* value	Mutation	*P* value	*P*_dominance_	*P*_recessive_	Wild	Heterozygous	*P* value	Mutation	*P* value	*P*_dominance_	*P*_recessive_
**Age**			0.789		0.528	0.976	0.379			0.150		0.135	0.124	0.632			0.106		0.612	0.119	0.942			0.798		0.513	0.965	0.470			0.374		0.701	0.377	0.885
Age ≤60	149	247		86				30	177		276				219	213		46				251	194		34				247	185		47			
Age >60	36	56		25				12	41		64				63	43		11				60	49		6				55	50		12			
**Gender**			0.082		0.756	0.215	0.190			**0.043**		0.124	0.074	0.656			0.637		0.311	0.939	0.231			0.176		0.803	0.254	0.572			0.542		**0.032**	0.824	**0.017**
Female	70	139		40				12	99		139				118	102		28				123	110		15				127	105		16			
Male	115	164		71				30	119		201				164	154		29				188	133		25				175	130		43			
**T stage**			0.723		0.489	0.617	0.551			0.056		0.264	0.142	0.537			0.106		0.783	0.205	0.442			0.557		0.657	0.741	0.540			0.763		0.480	0.970	0.423
1-2	19	33		7				9	14		37				25	29		5				33	20		7				34	21		5			
3-4	20	40		11				5	26		40				38	24		9				37	28		6				40	22		9			
**N stage**			0.370		0.926	0.463	0.578			0.485		0.484	0.457	0.820			0.979		0.165	0.652	0.154			0.170		0.476	0.162	0.730			0.577		0.468	0.463	0.554
Negative	7	19		3				4	9		16				15	13		1				12	14		3				14	11		4			
Positive	37	65		17				11	40		69				56	48		15				67	43		10				67	41		12			
**M stage**			0.572		0.577	0.522	0.733			0.383		0.543	0.457	0.861			0.291		0.213	0.666	0.100			0.646		0.864	0.649	0.951			0.237		0.358	0.180	0.526
Negative	24	36		10				8	22		41				35	24		11				36	29		6				42	22		7			
Positive	37	67		20				10	44		70				58	56		10				66	46		10				61	47		16			
**Clinical stage**			0.520		0.797	0.645	0.548			0.518		0.511	0.502	0.817			0.311		0.759	0.341	0.984			0.514		0.629	0.475	0.752			0.724		0.891	0.811	0.812
I-II	5	12		2				1	7		12				7	10		2				9	9		2				10	8		2			
III-IV	56	94		28				17	59		102				86	73		19				94	68		14				94	63		21			
**Smoking**			0.107		0.715	0.155	0.671			0.840		0.775	0.790	0.833			0.880		0.879	0.858	0.910			**0.034**		0.390	**0.033**	0.709			0.112		0.138	0.486	**0.045**
No	31	68		17				10	39		68				56	48		12				53	52		10				59	49		9			
Yes	30	39		14				8	28		47				39	35		9				51	26		6				46	23		14			
**Drinking**			0.451		0.572	0.423	0.795			0.783		0.689	0.894	0.269			0.925		0.781	0.986	0.748			0.959		0.378	0.744	0.374			0.948		0.098	0.476	0.085
No	46	86		25				14	50		94				75	66		16				81	61		14				85	58		15			
Yes	15	21		6				4	17		21				20	17		5				23	17		2				20	14		8			
**Family history of cancer**			0.471		0.974	0.557	0.739			0.907		0.607	0.699	0.438			0.719		0.452	0.578	0.502			0.782		0.573	0.953	0.510			0.590		0.835	0.597	0.943
No	51	92		26				16	58		96				82	70		17				89	67		12				88	63		19			
Yes	10	13		5				2	8		18				12	12		4				15	10		3				16	9		3			
**Pathological type**			0.754		**0.040**	0.389	**0.029**			0.523		0.644	0.568	0.986			0.198		0.067	0.090	0.095			0.614		0.538	0.656	0.476			0.617		0.596	0.686	0.538
small cell cancer	14	28		15				7	17		33				34	20		3				27	25		4				29	20		8			
squamous carcinoma	14	20		3				3	13		21				15	15		7				19	13		5				22	11		4			
adenocarcinoma	29	54		12				7	33		56				41	45		9				53	35		7				50	38		8			

**Table 5:** The interaction of ERCC1 and ERCC5 polymorphisms in the risk of lung cancer

		ERCC1 rs735482		ERCC1 rs11615		ERCC1 rs3212986		ERCC5 rs4771436		ERCC5 rs1047768		
		CC	CA+AA	GG	GA+AA	AA	CA+CC	GG	GT+TT	CC	CT+TT	
**ERCC1 rs735482**												
CC	Case/Control	/	/	/	/	/	/	/	/	/	/	
	OR (95%CI)	/	/	/	/	/	/	/	/	/	/	
CA+AA	Case/Control	/	/	/	/	/	/	/	/	/	/	
	OR (95%CI)	/	/	/	/	/	/	/	/	/	/	
		/		/		/		/		/		
**ERCC1 rs11615**												
GG	Case/Control	27/71	87/154	/	/	/	/	/	/	/	/	
	OR (95%CI)	1 (Ref)	1.21 (0.83,1.76)	/	/	/	/	/	/	/	/	
GA+AA	Case/Control	4/9	78/166	/	/	/	/	/	/	/	/	
	OR (95%CI)	0.96 (0.29,3.21)	0.69 (0.18,2.56)	/	/	/	/	/	/	/	/	
		P=0.574		/		/		/		/		
**ERCC1 rs3212986**												
AA	Case/Control	0/0	21/36	21/36	0/0	/	/	/	/	/	/	
	OR (95%CI)	1 (Ref)	1.16 (0.65,2.07)	1 (Ref)	1.18 (0.65,2.14)	/	/	/	/	/	/	
CA+CC	Case/Control	31/78	144/281	94/185	84/174	/	/	/	/	/	/	
	OR (95%CI)	0.76 (0.48,1.21)	NA	1.04 (0.73,1.50)	NA	/	/	/	/	/	/	
		0.608		0.583		/		/		/		
**ERCC5 rs4771436**												
GG	Case/Control	1/6	15/18	6/16	10/8	1/1	15/23	/	/	/		/
	OR (95%CI)	1 (Ref)	1.64 (0.80,3.36)	1 (Ref)	2.75 (1.04,7.25)	1 (Ref)	1.33 (0.68,2.63)	/	/	/		/
GT+TT	Case/Control	29/73	149/299	107/206	75/166	20/34	161/333	/	/	/		/
	OR (95%CI)	0.78 (0.48,1.25)	0.26 (0.03,2.24)	1.14 (0.80,1.64)	2.60 (0.07,1.02)	1.25 (0.70,2.25)	1.28 (0.07,23.59)	/	/	/		/
		0.247		0.054		0.868		/		/		
**ERCC5 rs1047768**												
CC	Case/Control	5/7	18/29	12/23	11/13	3/5	20/31	0/0	23/36	/		/
	OR (95%CI)	1 (Ref)	1.19 (0.64,2.22)	1 (Ref)	1.78 (0.76.4.16)	1 (Ref)	1.31 (0.72,2.37)	1 (Ref)	1.32 (0.75,2.30)	/		/
CT+TT	Case/Control	26/72	147/288	103/201	74/159	18/31	158/324	16/24	159/332	/		/
	OR (95%CI)	0.69 (0.42,1.13)	1.70 (0.43,6.77)	1.09 (0.76,1.57)	0.57 (0.19,1.77)	1.23 (0.66,2.26)	0.76 (0.15,3.99)	1.38 (0.71,2.67)	NA	/		/
		0.454		0.332		0.745		0.345		/		
												

**Table 6:** Epistatic effect analysis of ERCC1 and ERCC5 polymorphisms with lung cancer risk

SNP1	SNP2	SNP3	CON vs CA	
			*P* value	OR (95%CI)
rs735482	rs11615	rs3212986	0.897	1.04 (0.56,1.92)
rs735482	rs11615	rs4771436	0.323	1.39 (0.72,2.69)
rs735482	rs11615	rs1047768	0.307	1.34 (0.77,2.33)
rs735482	rs3212986	rs4771436	0.333	1.39 (0.72,2.68)
rs735482	rs3212986	rs1047768	0.337	1.31 (0.75,2.29)
rs11615	rs3212986	rs4771436	0.345	1.37 (0.71,2.64)
rs11615	rs3212986	rs1047768	0.382	1.28 (0.74,2.23)
rs3212986	rs4771436	rs1047768	0.339	1.31 (0.75,2.29)

**Table 7:** Haplotype-base risk prediction of SNPs in ERCC1 and ERCC5 genes for lung cancer

Gene	SNPs	Haplotype	Model^a^	F value	T value	OR	P value
ERCC1	rs4771436-rs1047768	TC	Unadjusted	0.297	0.011	0.986	0.917
			Adjusted	0.297	0.139	1.070	0.709
	rs4771436-rs1047768	GT	Unadjusted	0.271	0.103	1.050	0.748
			Adjusted	0.271	0.056	0.954	0.813
	rs4771436-rs1047768	TT	Unadjusted	0.430	0.021	0.982	0.885
			Adjusted	0.430	0.015	0.979	0.903
ERCC5	rs735482-rs11615	CA	Unadjusted	0.021	0.027	1.090	0.869
			Adjusted	0.021	0.024	1.120	0.877
	rs735482-rs11615	AA	Unadjusted	0.230	0.059	1.040	0.808
			Adjusted	0.230	0.301	0.893	0.583
	rs735482-rs11615	CG	Unadjusted	0.417	0.546	0.909	0.460
			Adjusted	0.417	0.056	0.957	0.813
	rs735482-rs11615	AG	Unadjusted	0.332	0.245	1.070	0.621
			Adjusted	0.332	0.484	1.140	0.486
ERCC5	rs3212986-rs11615	CA	Unadjusted	0.253	0.043	1.030	0.835
			Adjusted	0.253	0.307	0.896	0.580
	rs3212986-rs11615	AG	Unadjusted	0.311	0.034	1.020	0.855
			Adjusted	0.311	1.030	0.030	0.863
	rs3212986-rs11615	CG	Unadjusted	0.437	0.131	0.955	0.718
			Adjusted	0.437	0.117	1.060	0.732

## References

[1] Xue Q, Liu Z, Feng Z, Xu Y, Zuo W, Wang Q, Gao T, Zeng J, Hu X, Jia F (2020). Penfluridol: An antipsychotic agent suppresses lung cancer cell growth and metastasis by inducing G0/G1 arrest and apoptosis. Biomedicine & pharmacotherapy = Biomedecine & pharmacotherapie.

[2] Lorenzo-González M, Ruano-Ravina A, Torres-Durán M, Kelsey KT, Provencio M, Parente-Lamelas I, Leiro-Fernández V, Vidal-García I, Castro-Añón O, Martínez C (2019). Residential radon, genetic polymorphisms in DNA damage and repair-related. Lung cancer (Amsterdam, Netherlands).

[3] Wang L, Wang LL, Shang D, Yin SJ, Sun LL, Wang XY, Ji HB (2019). Gene polymorphism of DNA repair gene X-ray repair cross complementing group 1 and xeroderma pigmentosum group D and environment interaction in non-small-cell lung cancer for Chinese nonsmoking female patients. The Kaohsiung journal of medical sciences.

[4] Yu T, Xue P, Cui S, Zhang L, Zhang G, Xiao M, Zheng X, Zhang Q, Cai Y, Jin C (2018). Rs3212986 polymorphism, a possible biomarker to predict smoking-related lung cancer, alters DNA repair capacity via regulating ERCC1 expression. Cancer medicine.

[5] Anoushirvani AA, Aghabozorgi R, Ahmadi A, Arjomandzadegan M, Khalili S, Sahraei M, Fereydouni T, Khademi Z (2019). The Relationship Between rs3212986C>A Polymorphism and Tumor Stage in Lung Cancer Patients. Cureus.

[6] Zhou RM, Li Y, Wang N, Zhang XJ, Dong XJ, Guo W (2006). Correlation of XPC Ala499Val and Lys939Gln polymorphisms to risks of esophageal squamous cell carcinoma and gastric cardiac adenocarcinoma. Ai zheng = Aizheng = Chinese journal of cancer.

[7] Jin MH, Oh DY (2019). ATM in DNA repair in cancer. Pharmacology & therapeutics.

[8] Kay J, Thadhani E, Samson L, Engelward B (2019). Inflammation-induced DNA damage, mutations and cancer. DNA repair.

[9] Cavic M, Spasic J, Krivokuca A, Boljevic I, Kuburovic M, Radosavljevic D, Jankovic R (2019). TP53 and DNA-repair gene polymorphisms genotyping as a low-cost lung adenocarcinoma screening tool. Journal of clinical pathology.

[10] Chaszczewska-Markowska M, Kosacka M, Chryplewicz A, Dyła T, Brzecka A, Bogunia-Kubik K (2019). ECCR1 and NFKB2 Polymorphisms as Potential Biomarkers of Non-small Cell Lung Cancer in a Polish Population. Anticancer research.

[11] Li W, Zhang M, Huang C, Meng J, Yin X, Sun G (2019). Genetic variants of DNA repair pathway genes on lung cancer risk. Pathology, research and practice.

[12] Martínez-Ramírez OC, Pérez-Morales R, Castro-Hernández C, Gonsebatt ME, Casas-Ávila L, Valdés-Flores M, Petrosyan P, de León-Suárez VP, Rubio J (2019). Association of the Promoter Methylation and the rs12917 Polymorphism of MGMT with Formation of DNA Bulky Adducts and the Risk of Lung Cancer in Mexican Mestizo Population. DNA and cell biology.

[13] Zhang H, Li Y, Guo S, Wang Y, Wang H, Lu D, Wang J, Jin L, Jiang G, Wu J (2020). Effect of ERCC2 rs13181 and rs1799793 polymorphisms and environmental factors on the prognosis of patients with lung cancer. American journal of translational research.

[14] Bever KM, Le DT (2018). DNA repair defects and implications for immunotherapy. The Journal of clinical investigation.

[15] Sang L, Lv Z, Sun LP, Xu Q, Yuan Y (2018). Impact of SNP-SNP interactions of DNA repair gene ERCC5 and metabolic gene GSTP1 on gastric cancer/atrophic gastritis risk in a Chinese population. World journal of gastroenterology.

[16] Liu Y, Hu Y, Zhang M, Jiang R, Liang C (2018). Polymorphisms in ERCC2 and ERCC5 and Risk of Prostate Cancer: A Meta-Analysis and Systematic Review. Journal of Cancer.

[17] Kiyohara C, Yoshimasu K (2007). Genetic polymorphisms in the nucleotide excision repair pathway and lung cancer risk: a meta-analysis. International journal of medical sciences.

[18] Zhou RM, Niu CX, Wang N, Liu L, Huang X, Chen ZF, Huo XR, Hao YL, Li Y (2016). XPG Gene Polymorphisms and the Risk of Gastric Cardia Adenocarcinoma. Genetic testing and molecular biomarkers.

[19] Liu ZQ, Chen GG, Sun RL, Chen C, Lu MY, Guan LF, Chi XL, Jian YQ, Zhu X, Liu RQ (2018). XPG rs873601 G>A contributes to uterine leiomyoma susceptibility in a Southern Chinese population. Bioscience reports.

[20] Qi L, Yu HQ, Zhang Y, Ding LJ, Zhao DH, Lv P, Wang WY, Xu Y (2017). A Comprehensive Meta-analysis of Genetic Associations Between Key Polymorphic Loci in DNA Repair Genes and Glioma Risk. Molecular neurobiology.

[21] Jung SW, Park NH, Shin JW, Park BR, Kim CJ, Lee JE, Shin ES, Kim JA, Chung YH (2012). Polymorphisms of DNA repair genes in Korean hepatocellular carcinoma patients with chronic hepatitis B: possible implications on survival. Journal of hepatology.

[22] Bai Y, Xu L, Yang X, Hu Z, Yuan J, Wang F, Shao M, Yuan W, Qian J, Ma H (2007). Sequence variations in DNA repair gene XPC is associated with lung cancer risk in a Chinese population: a case-control study. BMC cancer.

[23] Kutob L, Schneider F (2020). Lung Cancer Staging. Surgical pathology clinics.

[24] Duran G, Aguin S, Cruz R, Barros F, Giraldez JM, Bernardez B, Lopez-Lopez R, Carracedo A, Lamas MJ (2019). Association of GSTP1 and ERCC1 polymorphisms with toxicity in locally advanced head and neck cancer platinum-based chemoradiotherapy treatment. Head & neck.

[25] Borchiellini D, Etienne-Grimaldi MC, Bensadoun RJ, Benezery K, Dassonville O, Poissonnet G, Llorca L, Ebran N, Formento P, Chateau Y (2017). Candidate apoptotic and DNA repair gene approach confirms involvement of ERCC1, ERCC5, TP53 and MDM2 in radiation-induced toxicity in head and neck cancer. Oral oncology.

[26] Hui EP, Ma BB, Chan KC, Chan CM, Wong CS, To KF, Chan AW, Tung SY, Ng WT, Cheng AC (2015). Clinical utility of plasma Epstein-Barr virus DNA and ERCC1 single nucleotide polymorphism in nasopharyngeal carcinoma. Cancer.

[27] Michiels S, Danoy P, Dessen P, Bera A, Boulet T, Bouchardy C, Lathrop M, Sarasin A, Benhamou S (2007). Polymorphism discovery in 62 DNA repair genes and haplotype associations with risks for lung and head and neck cancers. Carcinogenesis.

[28] Xu Q, Yuan Y, Sun LP, Gong YH, Xu Y, Yu XW, Dong NN, Lin GD, Smith PN, Li RW (2009). Risk of gastric cancer is associated with the MUC1 568 A/G polymorphism. International journal of oncology.

[29] Motegi A, Masutani M, Yoshioka KI, Bessho T (2019). Aberrations in DNA repair pathways in cancer and therapeutic significances. Seminars in cancer biology.

[30] Zhang X, Crawford EL, Blomquist TM, Khuder SA, Yeo J, Levin AM, Willey JC (2016). Haplotype and diplotype analyses of variation in ERCC5 transcription cis-regulation in normal bronchial epithelial cells. Physiological genomics.

[31] Huang Y, Meng C, Long W, Liu Y, Liu Y, Yang J, Yan Z, Yu D, Xiao S (2019). [XP gene polymorphisms and haplotypes with genetic susceptibility to lung cancer]. Wei sheng yan jiu = Journal of hygiene research.

[32] Zhang C, Liao Z, Yu G, Huang W, Song X (2017). Study on association between ERCC5 single nucleotide polymorphism and susceptibility to esophageal cancer. Journal of BUON: official journal of the Balkan Union of Oncology.

[33] Hussain SK, Mu LN, Cai L, Chang SC, Park SL, Oh SS, Wang Y, Goldstein BY, Ding BG, Jiang Q (2009). Genetic variation in immune regulation and DNA repair pathways and stomach cancer in China. Cancer epidemiology, biomarkers & prevention: a publication of the American Association for Cancer Research, cosponsored by the American Society of Preventive Oncology.

[34] Minina VI, Bakanova ML, Soboleva OA, Ryzhkova AV, Titov RA, Savchenko YA, Sinitsky MY, Voronina EN, Titov VA, Glushkov AN (2019). Polymorphisms in DNA repair genes in lung cancer patients living in a coal-mining region. European journal of cancer prevention: the official journal of the European Cancer Prevention Organisation (ECP).

[35] Shao L, Zuo X, Yang Y, Zhang Y, Yang N, Shen B, Wang J, Wang X, Li R, Jin G (2019). The inherited variations of a p53-responsive enhancer in 13q12.12 confer lung cancer risk by attenuating TNFRSF19 expression. Genome biology.

